# Impaired Decision Making and Loss of Inhibitory-Control in a Rat Model of Huntington Disease

**DOI:** 10.3389/fnbeh.2016.00204

**Published:** 2016-10-26

**Authors:** Nicole El Massioui, Charlotte Lamirault, Sara Yagüe, Najia Adjeroud, Daniel Garces, Alexis Maillard, Lucille Tallot, Libo Yu-Taeger, Olaf Riess, Philippe Allain, Huu Phuc Nguyen, Stephan von Hörsten, Valérie Doyère

**Affiliations:** ^1^Institut des Neurosciences Paris-Saclay (Neuro-PSI), UMR 9197, Centre National de la Recherche Scientifique (CNRS) Université Paris Sud, Université Paris SaclayOrsay, France; ^2^Neuropsychological Unit, Department of Neurology, CHU AngersFrance; ^3^The Graduate Center, City University of New York (CUNY)New York, NY, USA; ^4^Institute of Medical Genetics and Applied Genomics, University of TuebingenTuebingen, Germany; ^5^Center for Rare Diseases, University of TuebingenTuebingen, Germany; ^6^Experimental Therapy, Franz Penzoldt Center, Friedrich-Alexander University, Erlangen-NürnbergGermany

**Keywords:** impulsivity, decision-making, Huntington disease, gambling task, delay discounting, DRL

## Abstract

Cognitive deficits associated with Huntington disease (HD) are generally dominated by executive function disorders often associated with disinhibition and impulsivity/compulsivity. Few studies have directly examined symptoms and consequences of behavioral disinhibition in HD and its relation with decision-making. To assess the different forms of impulsivity in a transgenic model of HD (tgHD rats), two tasks assessing cognitive/choice impulsivity were used: risky decision-making with a rat gambling task (RGT) and intertemporal choices with a delay discounting task (DD). To assess waiting or action impulsivity the differential reinforcement of low rate of responding task (DRL) was used. In parallel, the volume as well as cellular activity of the amygdala was analyzed. In contrast to WT rats, 15 months old tgHD rats exhibited a poor efficiency in the RGT task with difficulties to choose advantageous options, a steep DD curve as delays increased in the DD task and a high rate of premature and bursts responses in the DRL task. tgHD rats also demonstrated a concomitant and correlated presence of both action and cognitive/choice impulsivity in contrast to wild type (WT) animals. Moreover, a reduced volume associated with an increased basal cellular activity of the central nucleus of amygdala indicated a dysfunctional amygdala in tgHD rats, which could underlie inhibitory dyscontrol. In conclusion, tgHD rats are a good model for impulsivity disorder that could be used more widely to identify potential pharmacotherapies to treat these invasive symptoms in HD.

## Introduction

Impulsiveness refers to the tendency to engage in inappropriate or maladaptive behaviors, without weighing consequences of actions. Impulse control disorders and impulsiveness are diagnostic criteria for several neuropsychiatric pathologies (Coles et al., 1997; Hucker, 1997; Johnson et al., 2000). Poor risk assessment and altered behavioral inhibition are also frequently encountered personality traits in some neurodegenerative diseases, such as Parkinson’s (PD; Antonini et al., 2011; Bugalho and Oliveira-Maia, 2012; Weintraub et al., 2015) or Huntington’s disease (HD Beglinger et al., 2008; Kalkhoven et al., 2014).

At a neurobiological level, self-control is thought to emerge from the dynamic interaction between an impulsive system, in which the amygdala is a neural structure critical in processing the affective and emotional signals of immediate outcomes, and a reflective system, in which the prefrontal (PFC) and orbitofrontal cortices are crucial in triggering the affective and emotional signals of long-term outcomes (Bechara, 2005). In healthy humans, a high level of impulsivity in a delay discounting task (DD) was associated with higher amygdala activation for winning immediate rewards (Ludwig et al., 2015). Moreover, trait impulsivity was positively correlated with the level of activity in response to reward cues in the anterior cingulate cortex (ACC) and amygdala (Kerr et al., 2015). In healthy rats, disconnection of the medial PFC and basolateral amygdala induces high level of impulsivity assessed in a DD task (Churchwell et al., 2009). Similarly, patients with selective amygdala damage (Urbach-Wiethe syndrome) have lower scores in decision-making under ambiguity and under risk (Brand et al., 2007), and lack of autonomic responses to reward and punishment used as “somatic markers” to guide future decision (Gupta et al., 2011).

Huntington disease (HD) is an autosomal dominantly inherited and progressive neurodegenerative disorder caused by an expanded CAG repeat of variable length in exon 1 of the gene encoding the protein huntingtin. HD causes degeneration of the medium spiny neurons of the striatum, but also neuronal death in the cerebral cortex and limbic structures. In HD patients, several studies found reduced amygdala volume (Thieben et al., 2002; Rosas et al., 2003; Depue et al., 2014; Dogan et al., 2014) associated with increased amygdala activity and disease burden (Dogan et al., 2014). Non-motor deficits associated with HD are generally dominated by executive function disorders (Watkins et al., 2000; Minati et al., 2011; Holl et al., 2013), often leading to decision-making impairments (for review see Stout et al., 2011). This dysexecutive syndrome is often associated with other behavioral consequences such as disinhibition (often concomitantly with apathy) and impulsivity/compulsivity (Duff et al., 2007; Beglinger et al., 2008; Reedeker et al., 2011). Few studies have directly examined symptoms and consequences of behavioral disinhibition in HD (Busemeyer and Stout, 2002; Stout et al., 2011; Holl et al., 2013) and only two studies have assessed risky decision-making abilities (Adjeroud et al., 2015) and levels of impulsivity in a transgenic rat model of HD (Manfré et al., 2016).

In the present study, as a first attempt to relate amygdala dysfunction and impulse control deficits in HD, we assessed in a within-subject design both risky decision-making and impulsivity as well as amygdala basal activity in early-symptomatic transgenic model of HD (tgHD) rats, a transgenic rodent model of HD carrying 51 CAG repeats under the control of the endogenous rat huntingtin promoter (Holzmann et al., 1998). Similar to the late-onset form of the human disease, tgHD rats exhibit adult-onset and slowly progressive phenotypes with impairments in motor, cognitive and affective behavior, as well as nuclear inclusions and neuropil aggregates, striatal atrophy and enlarged ventricles (Von Hörsten et al., 2003). Cognitive/choice impulsivity was assessed through two tasks: risky decision-making with a rat gambling task (RGT) and intertemporal choices with a DD task, often presented as a decision between “smaller, sooner” and “larger, later” rewards. Motor/action impulsivity was measured through the differential reinforcement of low rate of responding task (DRL). Amygdala basal activity was assessed in the same animals by immediate early gene analysis (cFos).

## Materials and Methods

### Animals

The study was performed on naïve female rats from our breeding colony, with eight wild type (WT) and 10 homozygous tgHD (Von Hörsten et al., 2003). They were 15 months old at the beginning of the experiments. Animals were housed two or three per cage in a room with controlled temperature and humidity with a 12–12 h day-night cycle (lights on at 8 am). The experiments were performed during the “light” cycle, 5–6 days per week. The animals had free access to water, and were food restricted and maintained at 85% of their *ad libitum* weight. Rats were successively trained on a RGT, a DRL and a DD task. All experiments were conducted in accordance with the guidelines established by the European Communities Council Directive (2010/63/EU Council Directive Decree) for compliance and use of laboratory animals. The protocol was approved by the ethical committee Paris-Sud and Centre (CEEA N°59).

### Apparatus

Four operant boxes (31 cm × 25 cm × 31 cm; Coulbourn Instruments, Whitehall, PA, USA) in soundproofed ventilated chambers (background noise 65 dB) were controlled with a Graphic State program (Coulbourn Instruments, Whitehall, PA, USA). For the RGT, the left curved wall was equipped with five circular holes (2 cm in diameter, 2 cm from the floor), with the center one inactive. Each of the four other holes could be dimly illuminated with a white light-emitting diode. A food magazine was positioned on the right wall for food pellets delivery (45 mg Purified Rodent Diet, Bio-Serv). For the DRL task, the operant boxes were equipped with a retractable lever (4-cm from the floor) positioned 3 cm on the right side of the magazine, and a red light (4 lux) as a house light. For the DD task, the same configuration as for DRL was used, except that there were two retractable levers, one on each side of the food magazine.

### Rat Gambling Task (RGT)

In the RGT, adapted from Rivalan et al. (2009) and as described in Adjeroud et al. (2015), rats have to face four options reinforced by an immediate reward. They have to learn that two options are associated with a greater reward, but are disadvantageous in the long run due to higher penalties. These contingencies are arranged to create a conflicting situation between the reward size at each trial and the overall quantity of reward over the session.

#### Pretraining

After a 30-min session of magazine training (VI30), rats learned first to associate one nose-poke in one illuminated hole with the delivery of one food pellet. The hole remained illuminated until the rat collected the food reward. After 1 s of blackout, the next trial started with the illumination of a different hole chosen pseudo-randomly to ensure equal experience with all four options. This procedure continued daily until rats obtained 80 pellets in less than 60 min. The next day after reaching this criterion, rats learned to perform two consecutive nose-pokes in one illuminated hole to obtain one pellet, with the same learning criterion. Finally, the next phase consisted in associating two consecutive nose-pokes in one illuminated hole with the delivery of two food pellets. The criterion was reached when rats obtain 160 pellets in 60 min.

#### Gambling Task

Twenty-four hours after the last training session, rats could freely choose between the four simultaneously illuminated holes (A–D) during a 1-h test session, with each choice being associated with a particular outcome. For half of the animals in each group, choices A and B allowed the immediate delivery of two food pellets but could be followed by long, unpredictable time-outs (222 s and 444 s, respectively; disadvantageous choices) during which no choice could be made. The probability of time-outs was 50% for hole A and 25% for hole B. Choices C and D allowed the immediate delivery of a single food pellet but could be followed by shorter, unpredictable time-outs (12 s and 6 s respectively; advantageous choices) with either 25% probability for choice C or 50% probability for choice D. For the other half of the animals, the position of advantageous vs. disadvantageous choices was counterbalanced and holes A and B allowed making advantageous choices whereas holes C and D were associated with disadvantageous choices. The theoretical maximum gain was the same for advantageous choices, and five times higher than for disadvantageous choices. During the time-out period, the chosen hole remained illuminated (flashing light: 0.5 s on, 0.5 s off) to facilitate the association between each choice and its consequences. A 1-s termination of this light signaled the end of the trial. All four holes were then again illuminated, allowing the rat to make a new choice. The session lasted 60 min.

#### Analysis

The number of sessions to reach each criterion during pretraining was calculated. For the RGT session, the percentage of choices (two consecutive nose pokes) for each option and the percentage of advantageous choices were calculated per 10-min periods (six periods per session). Each animal was also categorized as low impulsive if performing >70% of advantageous choices and high impulsive if performing <25% of advantageous choices (Rivalan et al., 2009).

### Differential Reinforcement of Low Rates of Responding (DRL) Task

The DRL task was chosen to assess impulsive action defined as the inability to withhold responses (lack of behavioral control). In this task, the reward is contingent upon responses spaced *t* seconds or more from the previous response. If the time between two responses is less than *t* seconds, no reward is delivered and the timing contingency is reset.

Rats were first trained to press a lever to obtain food pellets under a continuous reinforcement schedule (CRF) until 70 reinforcements were earned in 30 min. Then, rats were trained in DRL 5 s for five daily sessions. For each session, the house light was illuminated and the lever was inserted into the chamber. The first lever-press was reinforced. Then, a lever-press that occurred after a minimum 5 s delay was reinforced by the delivery of one food pellet and the illumination of a cue light inside the food magazine during a 2 s period. No cue indicated that a premature response had been made other than the lack of reinforcement. Timing contingency was reset immediately after the pellet-delivery period or after a lever-press if occurring within 5 s from the preceding one. Sessions ended after 60 min or 200 reinforcements, whichever came first.

#### Analysis

For each DRL session, the ratio of reinforced to total responses (efficiency) was used as the performance index. We also categorized the animals based on impulsivity for action criterion, with high impulsive rats having a DRL ratio <0.1, and low impulsive rats having a DRL ratio >0.2 (Simon et al., 2013). Moreover, the cumulative frequency of lever pressing per each 1 s time bin (inter-response time, IRT) during the delay period was calculated on the last DRL session. The probability of responding within each 1 s IRT category was calculated, determined by dividing the number of responses that fall into one category by the number of opportunities that the subject had to respond in that category. The number of opportunities for any category equals the number of responses falling in the category in question, plus all responses with longer IRTs (Kramer and Rilling, 1970). The relative frequency of burst responses (0–1 s), premature responses (1–4 s) and timing errors were also calculated for WT and tgHD groups.

### Delay Discounting (DD) Task

The DD task was used to assess impulsive choice, defined as preference for small, immediate rewards over larger, delayed rewards. The task design was the same as in Manfré et al. (2016).

#### Training

Rats were first trained to press two levers to obtain food pellets under CRF until 50 reinforcements were earned in less than 30 min. On one session, responding upon the left lever was reinforced and on another session, responding on the right lever was reinforced.

Animals were first trained during eight sessions to discriminate between a small (1 pellet) and a large (3 pellets) reward associated with the left or the right lever (counterbalanced between rats within each group). Five blocks of 12 trials were run during each session. Each block started with two forced choices using one single lever (one trial being a forced choice of the lever associated with the smaller option and one being a forced choice of the lever associated with the larger option), and 10 free choices between the two levers. Each 60 s trial began with a 10 s illumination of the food magazine. A nose-poke into the magazine during this time window extinguished the light and triggered an extension of either a single lever (forced-choice trials) or both levers simultaneously (free-choice trials) for a maximum of 10 s. Once a lever was pressed and food delivered, both levers were retracted for the remainder of the trial.

#### DD Testing

Rats were then tested for 12 sessions. Each session and trials structure were the same as during the previous stage except that increasing delays were introduced between lever pressing and the large reward. Each block consisted of two forced-choice trials used to expose the rats to the delays in effect for that block, followed by 10 free-choice trials. The delay duration increased between each block of trials (0, 4, 8, 16, 32 s), but remained constant within each block and were reset across sessions.

#### Analysis

The averaged percentage of choice for the large reward was calculated for each delay and averaged for the last three sessions. For each delay, the animals’ choice was categorized as high impulsive for rats choosing <50% the delayed large reward, and low impulsive for rats choosing >75% the delayed large reward (Simon et al., 2013).

### cFos Labeling

Fifteen days after the end of the DD task, animals were perfused transcardially with 100 ml of 0.9% sodium chloride containing 5% heparine and 1% sodium nitrite, followed by 300 ml of cold 4% paraformaldehyde (4°C) in 0.1 M phosphate buffer (PB). Brains were removed, post-fixed for 24 h at 4°C in the same fixative, and immersed in a graded series of sucrose phosphate-buffered solutions (12%, 16% and 18%). Serial coronal sections (40 μm thick) were cut with a microtome and collected in anatomical series. Immunohistochemistry was performed on free-floating sections. Every 3rd brain sections from bregma −1.92 mm to −3 mm (total 8–10 sections per rat; Paxinos and Watson, 2004) were collected in 0.1 M PB solution for the immunohistochemisty. Tissue peroxidases were eliminated with 0.3% of H_2_O_2_ and methanol 20%, during a period of 30 min. Tissue was incubated for 48 h at 4°C in smooth agitation with a polyclonal primary antibody, rabbit anti-cFos (1:100, Santa Cruz Biotechnology, Santa Cruz, CA, USA), and then with an affinity-purified secondary biotinylated antibody, goat anti-rabbit (1:200, Vector Labs, BA-2000, Burlingame, CA, USA), for 90 min at room temperature. For magnification, we used preassembled biotin–avidin peroxidase complex according to the Vector Labs’ recommendations (ABC Elite, Vector Labs). Then, sections were exposed to DAB (3,3’-diaminobenzidine) solution until the tissue developed an intense staining, rinsed and mounted. Using ImageJ software (National Institute of Health, Rockville, MD, USA), c-Fos positive cells were quantified in BLA and CeA nuclei as a function of antero-posteriority from bregma: anterior part of amygdala: from −1.92 to −2.4; posterior part of amygdala: from −2.52 to −3. Numbers of labeled cells were then averaged for anterior or posterior coordinates.

Moreover, outline of amygdala subparts was done in reference to the brain atlas (Paxinos and Watson, 2004). Surfaces were then obtained with ImageJ software (National Institute of Health, Rockville, MD, USA) using a macro command. The volume of CeA and BLA was estimated by calculating the mean area of processed sections multiplied by the total number of sections for each structure (n*3) and multiplied by the thickness of each section (0.04 mm). Counting and outlining were done manually by the experimenter who was blinded to experimental conditions.

### Statistical Analyses

Contrast analyses of variance were performed using VAR3 statistical software (Rouanet et al., 1990) with a 0.05 threshold. Fisher exact test was used for comparing the proportion of impulsive rats between groups. To assess a possible reduction of amygdala volume and increased neuronal activity as in HD patients (see Dogan et al., 2014), we used unilateral Student *t* test for comparing the difference between amygdala nuclei volumes and the number of cFos labeled cells for WT and tgHD rats.

## Results

### Rat Gambling Task (RGT)

During pretraining, all rats learnt to perform two successive nose-pokes in the illuminated hole to get the reward. The number of sessions needed to reach the criterion decreased across the three steps (*F*_(2,32)_ = 67.36; *p* < 0.001) with no genotype effect (*F*_(1,16)_ = 2.81, ns) and no genotype × step interaction (*F* < 1).

**Figure 1:**
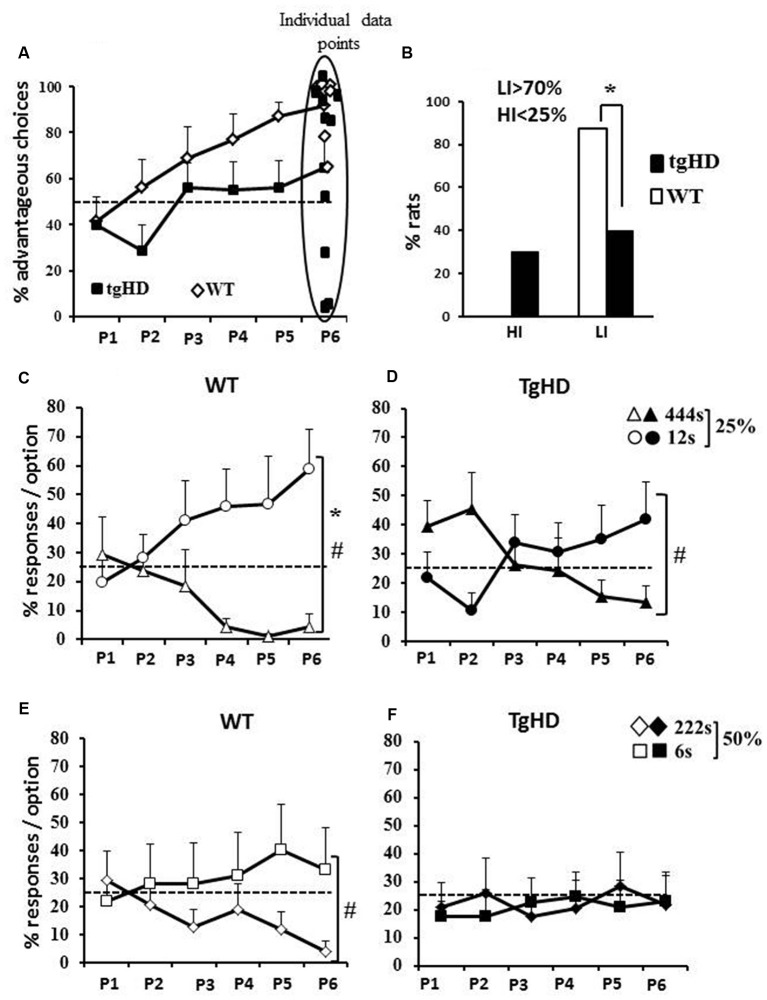
**Rat gambling task (RGT). (A)** Mean (+ SEM) percentage of advantageous choices by 10-min periods during the RGT session. Dotted line represents the random choice (50%); **(B)** Percentage of rats with high (<25% choices of advantageous options) or low (>75% of advantageous choices) impulsivity; Mean percentage of responses per option for wild type (WT) rats **(C,E)**, for transgenic model of HD (tgHD) rats **(D,F)** and by probability of penalties (25%: **C,D** or 50%: **E,F**). Dotted lines represent the random choice (25%). *Between-group difference; ^#^Time/period dependance. *p* < 0.05.

During the RGT session, both groups increased their rate of advantageous choices over the six 10 min periods (Figure 1A; WT: *F*_(5,35)_ = 6.57, *p* < 0.001; tgHD: *F*_(5,45)_ = 4.05, *p* < 0.01), with poorer performances for the tgHD rats. In effect, although no significant between group difference (*F*_(1,16)_ = 2.40, ns) and genotype × period interaction (*F*_(5,80)_ = 1.24, ns) were found, WT rats showed a progressive improvement of performance reaching 90% of correct choices during the last 10 min period (*p* = 0.001 from random on this last period), whereas tgHD rats reached only 64% of correct responses (*p* = 0.13 from random). Noticeably, the percentage of low impulsive rats was significantly lower for the tgHD group than for the WT group (*p* = 0.05; Figure 1B), which was however not reflected in different percentage of high impulsive rats (Figure 1B; *p* = 0.17).

An analysis per option indicates a differential improvement over the RGT session depending on the probability/penalty combination and the genotype. When considering the 25% probability of penalty, both groups of rats showed increasing performance for the “12 s timeout” option (Figure 1C; WT: *F*_(5,35)_ = 2.68, *p* < 0.05; Figure 1D; tgHD: *F*_(5,45)_ = 2.70, *p* < 0.05) and decreasing choices of the “444 s timeout” option (WT: *F*_(5,35)_ = 2.54, *p* = 0.05; tgHD: *F*_(5,45)_ = 3.01, *p* < 0.05), resulting in a Choice (444 s-25% vs. 12 s-25%) × Period interaction for both groups (WT, *F*_(5,35)_ = 2.82, *p* < 0.05; tgHD, *F*_(5,45)_ = 3.88, *p* < 0.01). However, there was a significant discrimination over all six test periods between the two holes for WT (*F*_(1,7)_ = 7.36, *p* < 0.05) and not for tgHD rats (*F* < 1). It is worth noting that tgHD rats responded more to the disadvantageous than to the advantageous option towards the beginning of the session (P2: *F*_(1,9)_ = 5.34, *p* < 0.05). Less robust changes in preference across training were observed for the 50% probability, especially for the tgHD rats. Although WT rats showed a significant choice × period interaction (*F*_(5,35)_ = 3.94, *p* < 0.01), they showed a decreasing preference of the “222 s timeout” (Figure 1E; *F*_(5,35)_ = 3.58, *p* < 0.05), but did not show an improvement of their preference for the hole associated with a 6 s timeout (*F*_(5,35)_ = 2.05, ns). In contrast, tgHD rats did not increase their preference for the option associated with a “6 s timeout” (*F*_(5,35)_ = 2.05, ns) nor decreased their preference for the option associated with “444 s timeout” (*F* < 1). They showed no discrimination between the two options (222 s and 6 s) and no choice × period interaction (Figure 1F; *F*s < 1). The results show that the percentage of occurrence of penalties is a key parameter in determining choice abilities, and that it influences particularly the transgenic animals.

### Differential Reinforcement of Low Rates of Responding (DRL)

**Figure 2:**
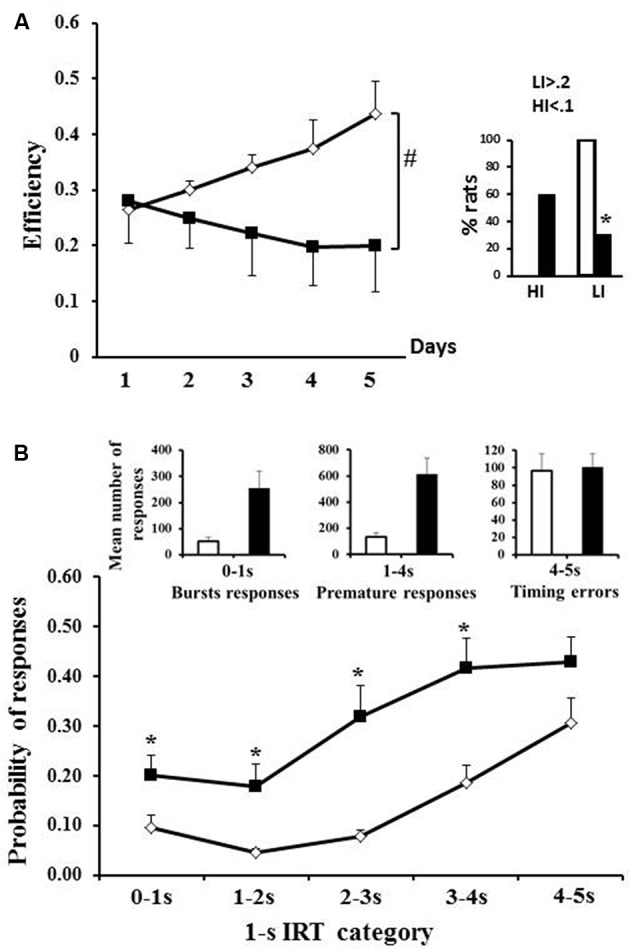
**Differential reinforcement of low rate of responding (DRL). (A)** Mean efficiency during the five sessions of DRL5 s for WT (open diamonds) and tgHD rats (black squares); the right histograms represent the percentage of rats with low (>0.2) or high impulsivity (<0.1; white column: WT; black columns: tgHD rats).** (B)** Histograms represent the mean number of burst responses, premature responses and timing errors. Lower curves represent the probability of responses for each 1 s category (cumulative frequency). *Between-group difference; ^#^Time/period dependance. *p* < 0.05.

Transgenic animals also showed poorer performance in the DRL task in which efficiency depends upon control of action (inhibition) and temporal processing. WT and tgHD rats diverged in efficiency with repeated training (Figure 2A, significant genotype × session interaction *F*_(4,64)_ = 6.41, *p* < 0.01). In effect, whereas WT rats progressively increased their efficiency with repeated training (*F*_(4,36)_ = 2.87, *p* < 0.05), tgHD rats showed the opposite results, i.e., a progressive decrease in efficiency (*F*_(4,28)_ = 3.47, *p* < 0.05). Compared to WT rats, a lower percentage of tgHD rats were scored as low impulsive (Figure 2A; *p* = 0.0002), with a tendency for a higher percentage of high impulsive rats (*p* = 0.059).

An analysis of the pattern of responses on the last training session (day 5) showed that, compared to WT, tgHD rats did more bursts of responses (Figure 2B; left histograms: *t* Student: *t* = 2.85, *p* = 0.005), and more premature responses (Figure 2B; central histograms: *t* Student: *t* = 3.01, *p* = 0.008), but showed similar number of timing errors (Figure 2B; right histograms, *t* Student: *t* = 0.18, *p* = 0.85). The analysis of probability of responding per 1 s IRT category (Figure 2B) showed that the probability of responding before the required delay (5 s) was more important in tgHD animals compared to WT (*F*_(1,16)_ = 11.17, *p* < 0.01) and increased as the delay increased (WT: *F*_(4,28)_ = 22.65, *p* < 0.001; tgHD: *F*_(4,36)_ = 13.12, *p* < 0.001). A group comparison for each delay also showed that tgHD animals responded more from the first to the fourth second of delay (0–1 s: *F*_(1,16)_ = 4.93, *p* < 0.05; 1–2 s: *F*_(1,16)_ = 6.88, *p* < 0.05; 2–3 s: *F*_(1,16)_ = 12.42, *p* < 0.01; 3–4 s: *F*_(1,16)_ = 10.34, *p* < 0.01). However, there was no more significant difference between both groups on the last IRT (*F*_(1,16)_ = 3.26, ns). Thus, the poorer efficiency of tgHD animals was due to their inability to inhibit their action after the reward delivery, rather than a poorer temporal processing.

### Delay Discounting (DD)

**Figure 3:**
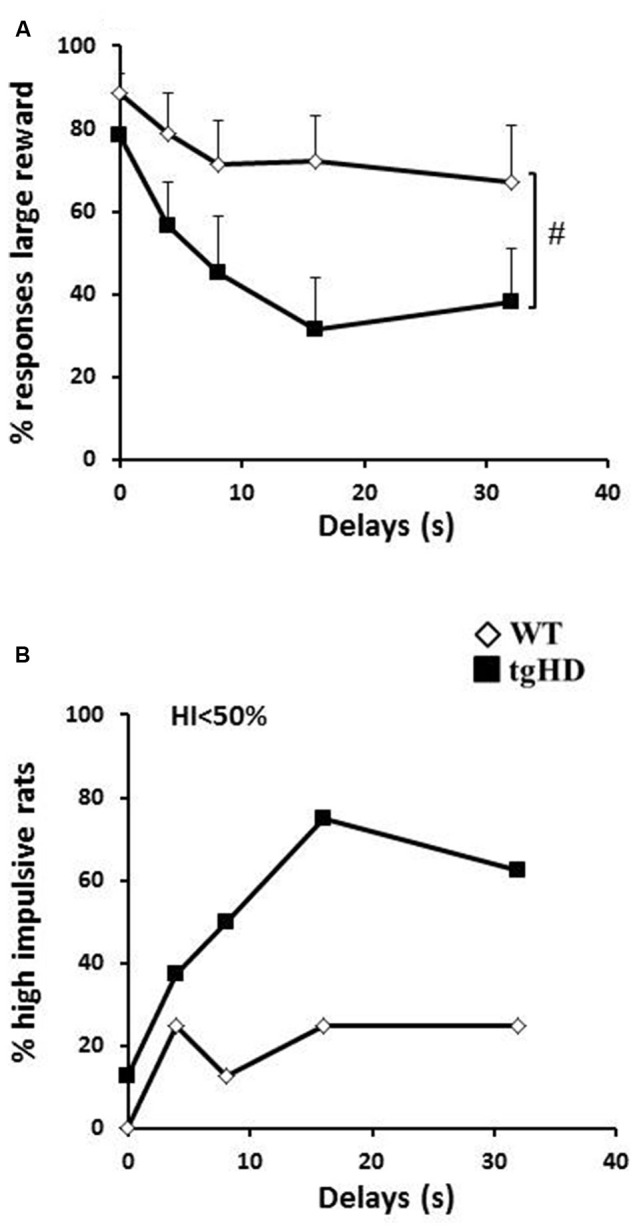
**Delay discounting (DD). (A)** Mean (+ SEM) percentage of responses to the large reward for the different delays (4, 8, 16 and 32 s. **(B)** Percentage of high impulsive rats (<50% choice of large reward). WT: open diamonds; tgHD: black squares. ^#^Time/period dependance.

Transgenic animals were also impaired in the DD task in which a delay was introduced between the action and reinforcement delivery. During training, two tgHD rats did not learn the initial discrimination between the two levers and were excluded. For the remaining rats, both groups similarly learnt to choose the large reward in more than 90% of free trials with no effect of session (*F*_(7,98)_ = 1.87, ns), no effect of genotype and no genotype × session interaction (*Fs* < 1). As expected, WT animals decreased their preference for the lever associated with the large reward when increasing delays were inserted between lever press and reward delivery (Figure 3A; *F*_(4,28)_ = 3.72, *p* < 0.05). This decrease was more pronounced in transgenic animals (*F*_(4,28)_ = 14.98, *p* < 0.001; significant genotype × delay interaction, *F*_(4,56)_ = 2.89, *p* < 0.05). As there was no omission of responses in all rats (except for one tgHD rat which showed 66.66% of responding to delays 8 and 16 s), the results indicate that animals (especially tgHD rats) shifted to the response for the immediate small reward. This greater difficulty for maintaining their choice for a large, but delayed, reward was also reflected by a tendency for a greater percentage of tgHD rats with a high impulsivity ratio as compared to WT rats, especially for the 16 s and 32 s delays (Figure 3B; 16 s: *p* = 0.06; 32 s: *p* = 0.09).

### Relationships Among Behavioral Variables

**Figure 4:**
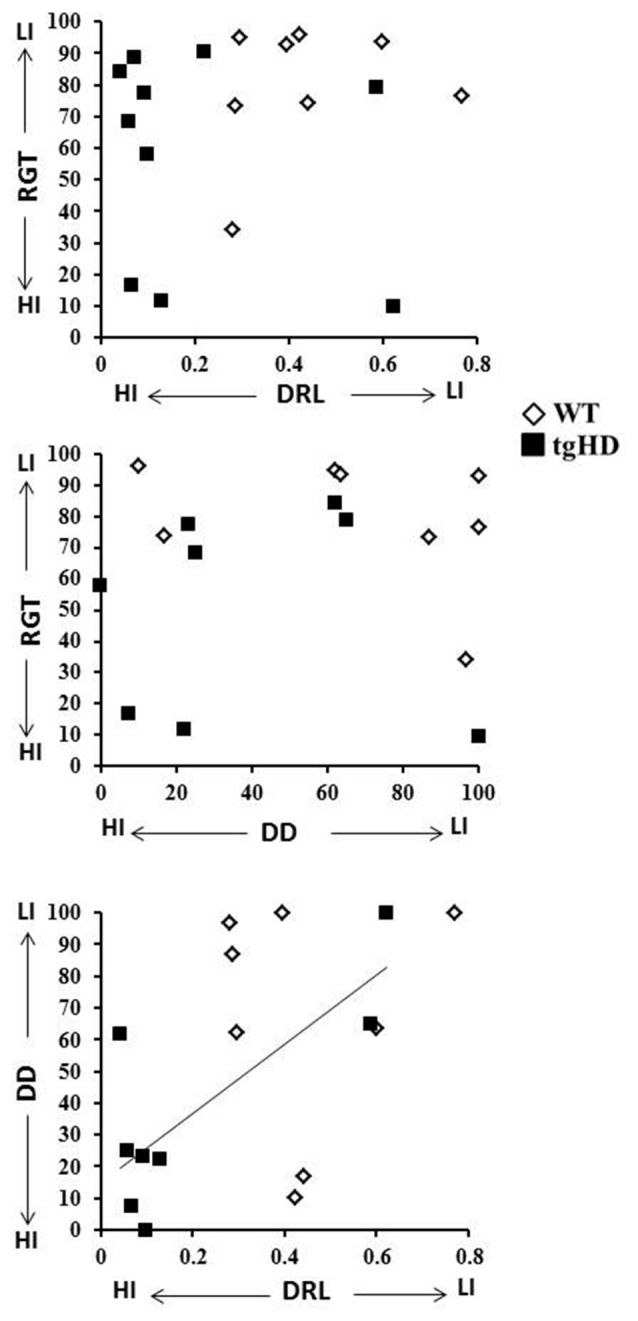
**Correlations.** This figure represents the diagram of correlation between RGT (mean percentage of advantageous choices) and efficiency on the last DRL session (upper diagram), between RGT and DD (percentage of choices for the 32 s delay; middle diagram) and between efficiency on the last DRL session and performance to the 32 s delay in the DD task (lower diagram). The line represents the significant correlation between DD and DRL for tgHD rats (open diamonds: WT rats; black squares: tgHD rats).

Pearson correlations were calculated to search for linear relationships between behavioral indices from the three tasks. Correlations measured for WT rats between RGT (mean percentage of advantageous choices for the entire session), DD (percentage of choice for the 32 s delay) and DRL (efficiency on the last session) performances showed no correlation for any of each pair of comparisons (Figure 4; *r* ranging from −0.30 to 0.25; *p*s > 0.05). For the tgHD rats, however, a significant correlation was found between DRL and DD (*r* = 0.777, *p* < 0.05), while neither of these tasks correlated with impulsivity in RGT (DRL/RGT: −0.22 and DD/RGT: −0.03; *p*s > 0.05).

### Amygdala Volume

**Figure 5:**
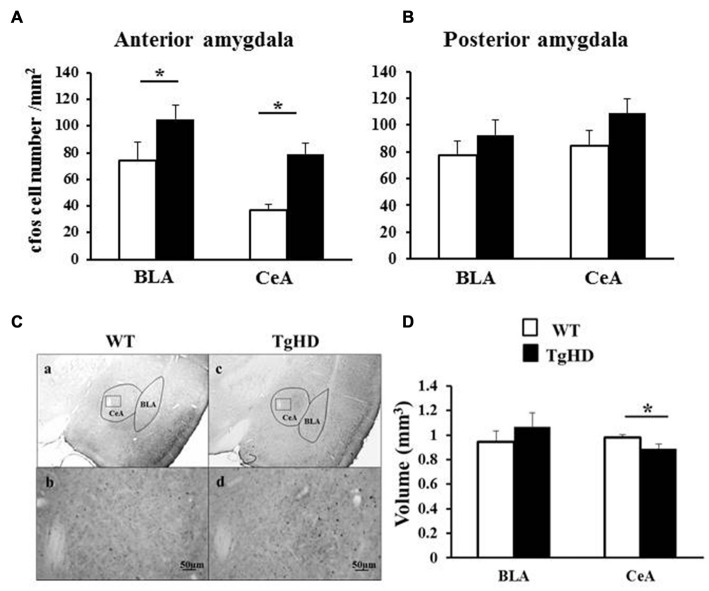
**Amygdala basal cellular activation.** Figures represent cFos labeled neurons in **(A)** the anterior amygdala (from −1.92 to −2.4 from bregma) and in **(B)** the posterior amygdala (from −2.52 to −3 from bregma). **(C)** Shows cFos labeling in WT **(a,b)** or tgHD **(c,d)** rats. The small squares in the CeA photos are expanded with a x20 magnification for WT rat **(b)** and TgHD rat **(d)**. Histograms in **(D)** represent the averaged volume of BLA and CeA in both genotypes (WT: white histograms; tgHD: black histograms). *p < 0.05.

As shown in Figure 5D, there was no genotype difference for the BLA volume (unilateral Student *t* test, *t*_(6)_ = −0.97, *p* = 0.18, ns). However, the CeA volume of tgHD rats was smaller than the CeA volume in WT animals (*t*_(6)_= 2.22, *p* = 0.033).

### cFos Expression in BLA and CeA

At the anterior level of the amygdala (Figures 5A,C), tgHD rats showed an increased basal activity both in BLA (*t*_(6)_ = 2.15, *p* = 0.037) and in CeA (*t*_(6)_ = 2.27, *p* = 0.031), compared to WT animals. In contrast, there was no genotype difference at the posterior level of the amygdala for BLA nor for CeA (Figure 5B; *p*s > 0.05).

## Discussion

The present findings show that 15 months old WT female rats were efficient decision makers, integrating outcomes of past decisions to determine expected reward values for each option, thus increasing progressively their performance to 90% of advantageous choices in the RGT. However, they were slightly less efficient when the probability of penalties was higher (50% compared to 25%). In contrast, 15 months old female tgHD rats appeared to be poor decision makers, showing difficulties to resist options that are immediately more rewarding, as indicated by their preference for the larger immediate reward option at the beginning of the RGT session, and thus failed to control their behavior in order to optimize their final gains. As WT animals, they showed worse performances for higher probability of penalties, up to an inability to learn in the 50% condition. The tgHD rats showed also poorer efficiency in the DRL task, with higher rates of premature and burst responses, and shifted more rapidly to the smaller immediate reward as the delay increased in the DD task, compared to WT rats. Interestingly, in contrast to WT animals, there was a positive correlation between cognitive (DD task) and action (DRL task) impulsivities for the tgHD animals. Finally, the same tgHD animals had a reduced volume of the CeA, as well as an increased basal cellular activity in the anterior part of the amygdala (BLA and CeA).

### Cognitive Impulsivity and Decision-Making in HD

In the RGT task, a risky decision-making task, WT rats were able to develop a strategy based on the consequences of their choices across trials, enabling them to earn a maximum total amount of reward at the end of the session. Similarly, in the DD task, WT rats were able to maintain their bias toward larger delayed gratification even though the delay between the instrumental response and the reward delivery increased, an index of high self-control. In contrast, the transgenic rats showed little efficiency during the whole RGT session (with a maximum of 64% of advantageous choices at the end of the session). At the beginning of the session, they showed more choices of the disadvantageous option with a large reward and long penalty when the probability of penalty was quite low (25%), suggesting a propensity for risk taking behavior. However, when the probability of penalty was high (50%) tgHD rats did not discriminate any more between disadvantageous and advantageous options, showing a poorer efficiency in higher risk conditions. In the DD task, tgHD animals showed a steep DD curve, shifting rapidly to the smaller and sooner reward as delay increased. In both RGT and DD tasks, tgHD rats’ performances indicate a higher level of cognitive/choice impulsivity than in WT animals. It is worth noting that tgHD rats had no difficulties learning the initial instrumental tasks, either pressing a lever to get the food reward for the DRL or the DD tasks, or putting their nose in holes to provoke food delivery in the RGT task. Their rate of learning was similar as for WT rats, indicating that tgHD rats had no problem in associative abilities, as already described in previous articles (Faure et al., 2011; Höhn et al., 2011; Fink et al., 2012). However, successful performance in the RGT and DD tasks requires flexibility in planning to account for various outcomes and memory to process incoming information and evaluate the risk–reward ratio. As in HD patients (Craufurd and Snowden, 2002; Allain et al., 2005, 2011), both cognitive impairments have been reported in tgHD animals. First, tgHD rats exhibit robust memory deficits in paradigms involving both hippocampal- and striatal-based memory systems (Zeef et al., 2012; Kirch et al., 2013). Second, tgHD rats show difficulties to change acquired behavior as the contingencies change (reversal tasks; Höhn et al., 2011; Fink et al., 2012), with more perseverative and/or premature responses (Kántor et al., 2006).

### Correlation Between Action and Cognitive Impulsivities in HD

In the DRL paradigm, impulsivity is characterized by the inability to withhold responses for a required amount of time. This task mostly involves two cognitive/behavioral abilities: first, behavioral inhibition or self-control (Barkley, 1997) indicated by bursts and premature responses; second, temporal discrimination ability, which allows to know when the time *t* has elapsed (Kramer and Rilling, 1970), indicated by timing errors. Burst responses, which immediately follow a rewarded lever-press, indicate perseverative responses induced by the failure to re-obtain an immediate feedback to their lever-presses. In the DRL task, tgHD rats exhibited higher rates of bursts and premature responses, thus showing an inability to withhold a response for a certain amount of time. In parallel, they showed a normal rate of timing errors, indicating a normal functioning of temporal estimation. Similarly, in a mouse model of HD, transgenic mice also showed a normal learning of critical temporal intervals (temporal accuracy). However, they showed a decreased temporal control over operant responding, reflecting a lack of inhibitory control (Balci et al., 2009). These deficits could account for the alteration of the decision processes of symptomatic tgHD animals.

In the current study, HD rats showed deficits in the three tasks, exhibiting alteration of the different types of impulsivity and in risky decision making. Correlation analyses between performances in the DRL, DD and RGT tasks indicate that these measures of different types of impulsivity were statistically independent in WT animals. In human subjects, impaired decision-making under risk seems to reflect a distinct psychological trait from other forms of impulsivity (Winstanley, 2011). Rodent or human studies, using similar behavioral tasks in both species, also report no correlations between action impulsivity and choice impulsivity, indicating that both types of impulsivity are multifaceted in nature and could rely on distinct neurobiological mechanisms (Lejuez et al., 2003; Winstanley et al., 2004; Reynolds et al., 2006; Diergaarde et al., 2008; Broos et al., 2012; Simon et al., 2013). In contrast, a significant positive correlation between choice impulsivity and action impulsivity appears in tgHD animals, as measured in the DD and DRL tasks. This significant positive correlation may index a parallel deterioration of neurobiological substrates underlying both measures of impulsivity.

### Potential Neurobiological Substrates of Impulse Dyscontrol in HD

The present results showed an increased basal cellular activity in BLA and CeA in 15-month old homozygous tgHD rats compared to WT, associated with a volume reduction of the CeA and high levels of behavioral impulsivity. It also appeared that the anterior part of BLA and CeA was more active than the posterior part. The functional involvement of anterior amygdala (CeA and BLA) in the reduced self-control in HD may be through its possible modulatory role on striatal functioning via the CeA-nigro-lateral striatal dopaminergic (DA) pathway (Gonzales and Chesselet, 1990) or through the BLA-prefronto-ventral striatal pathway (McDonald, 1991).

Homozygous tgHD rats exhibit adult-onset, slowly progressive phenotypes with a dynamic process leading to region- and age-specific polyQ recruitment and aggregation (Von Hörsten et al., 2003; Cao et al., 2006; Nguyen et al., 2006; Kirch et al., 2013). High number of aggregation sites and aggregates were observed in structures such as the nucleus accumbens and substantia nigra pars compacta before they were detected in cortical areas or the caudate-putamen (Nguyen et al., 2006), suggesting that both CeA-nigro-lateral striatum and BLA-prefronto-ventral striatum circuits could be deteriorated. In HD patients, *in vivo* imaging demonstrates striatal shrinkage in magnetic resonance images (Von Hörsten et al., 2003), as well as a reduced mean total number of neurons in the striatum (Kántor et al., 2006) and a faster increase in ventricle volume with age (Blockx et al., 2011). Twelve months old tgHD rats show an alteration of synaptic plasticity of the prefronto-striatal pathway (Höhn et al., 2011) as well as a reduced surface of the CeA associated with reduced affective responses to motivational events (Faure et al., 2011). TgHD rats (11 months old) also show DA alterations indicated by enhanced TH levels in the dorsal and ventral striatum (Jahanshahi et al., 2010, 2013), in line with postmortem clinical data (Bird et al., 1980; Spokes, 1980).

The different facets of impulsivity have been found to be modulated by dopamine transmission (Cardinal et al., 2000; Robbins, 2002; Floresco et al., 2008), and associated with specific dopamine imbalances in (fronto)striatal circuitry (Cole and Robbins, 1987; van Gaalen et al., 2006; Diergaarde et al., 2008). For example, an altered D2 receptor expression in the prelimbic cortex has been shown to be correlated with choice impulsivity (Castellanos and Tannock, 2002; Simon et al., 2013) whereas baseline levels of action impulsivity have been related to D2 receptor expression in the nucleus accumbens (Neill and Herndon, 1978; Dalley et al., 2007; Simon et al., 2013). However, according to the model proposed by Bechara (2005), self-control emerges from the dynamic interaction between an impulsive system, in which the amygdala is a critical neural structure involved in triggering the affective and emotional signals of immediate outcomes, and a reflective system, in which the ventral prefrontal cortex (PFC) is crucial in processing the affective and emotional signals of long-term outcomes. In a DD paradigm, rats with inactivation and disconnection of the medial PFC and basolateral amygdala become more impulsive, affecting preference for smaller immediate over larger delayed rewards (Churchwell et al., 2009). Similarly, patients with selective amygdala damage (Urbach-Wiethe syndrome) have lower scores in both decisions under ambiguity and decisions under risk (Brand et al., 2007) or lack the autonomic responses to reward and punishment used as “somatic marker” type cues to guide future decision-making (Gupta et al., 2011). Lesions of the BLA before task acquisition slowed learning, but did not prevent the accurate decision making, whereas post-acquisition lesions persistently altered choice efficiency by favoring the disadvantageous options as in patients with amygdala disorders (Zeeb and Winstanley, 2011). In HD patients, beside cortico-striatal alterations, several studies found reduced amygdala volume (Thieben et al., 2002; Rosas et al., 2003; Depue et al., 2014; Dogan et al., 2014). Moreover, more pronounced amygdala atrophy is related to a higher amygdala activity and disease burden (Dogan et al., 2014).

In homozygous tgHD rats, recent studies found enhanced TH levels in the dorsal and ventral triatum (Jahanshahi et al., 2010, 2013), in line with postmortem clinical data (Bird et al., 1980; Spokes, 1980). Furthermore, a reduction in striatal D1-receptor density was found in 14 months old tgHD rats (Bode et al., 2008), as well as a decrease in striatal D1 and D2 receptor binding at the age of 24 months (Bauer et al., 2005; Von Hörsten et al., 2003). A reduction of serotonin-containing cells and an increase of dopamine-containing cells were also found in the dorsal raphe nucleus in both human and tgHD rat tissues, inducing a reduced level of serotonin expression in the medial PFC (Jahanshahi et al., 2013). All these results indicate amygdala-prefronto-striatal circuits’ dysfunction in HD patients and tgHD rats, with associated dopamine dysregulation. To better understand the significance of these results, one must also take into account the high number of aggregation sites and aggregates in structures such as the olfactory tubercle, the nucleus accumbens, thalamus and substantia nigra pars compacta substantially before they were detected in cortical areas or the caudate-putamen (Nguyen et al., 2006), providing a possible anatomical correlate to the early onset of impulsivity symptoms. As specific dopamine or serotonin imbalances in fronto-striatal circuitry are known to be associated with the two distinct measures of impulsive behavior (action and choice impulsivity; Diergaarde et al., 2008; see Jentsch and Taylor, 1999), we can speculate that the increased amygdala neuronal activity could participate in the enhanced DA striatal activity observed in tgHD rats, as well as in HD patients.

## Conclusion

To date, no rodent studies have tested in a within-subject design risky decision-making, impulsive action and impulsive choice to assess the multidimensional construct of impulsivity in healthy rats or in transgenic rat model of HD. It is worth noting that our results were obtained using female tgHD rats, in which a less severe course of the disease has been reported compared to male tgHD rats at the behavioral, physiological and neuropathological levels (Bode et al., 2008; Urbach et al., 2014). One could wonder now when impulsivity emerges in the course of the disease in tgHD male and female animals. In all, searching for concomitant choice and action impulsivity traits could allow a rapid diagnosis and treatment of potential invasive impulsive traits and altered decision-making in HD patients.

## Author Contributions

NEM, CL and VD: conception of the experiments, analyses and writing of the article. SY, NA, DG, AM, CL and LT: conducting experiments and analyses. LY-T, OR, HPN and SvH: creation of the rat model of HD, discussions, genotyping and histological analyses. PA: conception of the experiments.

## Conflict of Interest Statement

The authors declare that the research was conducted in the absence of any commercial or financial relationships that could be construed as a potential conflict of interest.
